# Study of the persistence and dynamics of recombinant mCherry‐producing *Yarrowia lipolytica* strains in the mouse intestine using fluorescence imaging

**DOI:** 10.1111/1751-7915.14178

**Published:** 2022-12-20

**Authors:** Catherine Madzak, Sabine Poiret, Sophie Salomé Desnoulez, Benoit Foligné, Frank Lafont, Catherine Daniel

**Affiliations:** ^1^ INRAE, AgroParisTech, Paris‐Saclay University, UMR 782 SayFood Thiverval‐Grignon France; ^2^ Univ. Lille, CNRS, INSERM, CHU Lille, Institut Pasteur de Lille, U1019 ‐ UMR 9017 – Center for Infection and Immunity of Lille Lille France; ^3^ Univ. Lille, CNRS, INSERM, CHU Lille, Institut Pasteur de Lille, US 41 ‐ UMS 2014 – PLBS Lille France; ^4^ Univ. Lille, INSERM, CHU Lille, U1286 ‐ Infinite ‐ Institute for Translational Research in Inflammation Lille France

## Abstract

*Yarrowia lipolytica* is a dimorphic oleaginous non‐conventional yeast widely used as a powerful host for expressing heterologous proteins, as well as a promising source of engineered cell factories for various applications. This microorganism has a documented use in Feed and Food and a GRAS (generally recognized as safe) status. Moreover, in vivo studies demonstrated a beneficial effect of this yeast on animal health. However, despite the focus on *Y. lipolytica* for the industrial manufacturing of heterologous proteins and for probiotic effects, its potential for oral delivery of recombinant therapeutic proteins has seldom been evaluated in mammals. As the first steps towards this aim, we engineered two *Y. lipolytica* strains, a dairy strain and a laboratory strain, to produce the model fluorescent protein mCherry. We demonstrated that both *Y. lipolytica* strains transiently persisted for at least 1 week after four daily oral administrations and they maintained the active expression of mCherry in the mouse intestine. We used confocal microscopy to image individual *Y. lipolytica* cells of freshly collected intestinal tissues. They were found essentially in the lumen and they were rarely in contact with epithelial cells while transiting through the ileum, caecum and colon of mice. Taken as a whole, our results have shown that fluorescent *Y. lipolytica* strains constitute novel tools to study the persistence and dynamics of orally administered yeasts which could be used in the future as oral delivery vectors for the secretion of active therapeutic proteins in the gut.

## Graphical abstract

Yarrowia lipolytica is a dimorphic oleaginous non‐conventional yeast widely used as a powerful host for expressing heterologous proteins. To study its potential for oral delivery of recombinant therapeutic proteins in mammals, we engineered two Y. lipolytica strains to produce the model fluorescent protein mCherry. We demonstrated that both Y. lipolytica strains transiently persisted in mice after four daily oral administrations and they maintained the active expression of mCherry in the mouse intestine. We used confocal microscopy and showed that Yarrowia cells were found essentially in the lumen and they were rarely in contact with epithelial cells while transiting through the ileum, caecum, and colon of mice. Taken as a whole, our results have shown that fluorescent Y. lipolytica strains constitute novel tools to study the persistence and dynamics of orally administered yeasts.

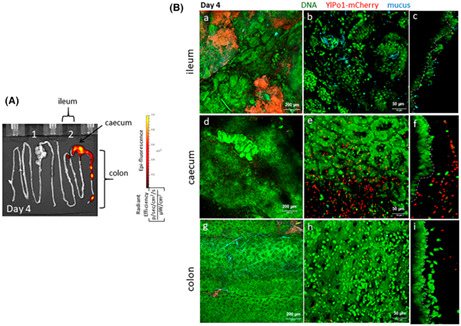

## INTRODUCTION

Yeasts combine the ease of use of microorganisms with a eukaryotic organization enabling post‐translational protein modification and suitable glycosylation, which makes them preferred over other microbial systems for the production of recombinant therapeutic proteins (Ivanova, 2021; Kim et al., 2015; Lee et al., 2015; Madhavan et al., 2021; Patra et al., 2021; Pöhlmann et al., 2013). *Y. lipolytica* is a dimorphic oleaginous yeast that has drawn the attention of industrialists in the 1950s and has since been recognized as a powerful host for the expression, secretion and surface display of heterologous proteins, as well as a promising source of engineered cell factories for a wide range of applications (Madzak, 2018, 2021a). This non‐conventional yeast is a biosafety level 1 microorganism with a documented use in food (International Dairy Federation/European Food and Feed Cultures Association) and a GRAS (generally recognized as safe) status (USA Food and Drug Administration) (Groenewald et al., 2014; Madzak, 2021a). The European Food and Safety Authority allowed in 2019 the use of *Y. lipolytica* biomass as a novel food for human consumptions thanks to its safety and nutritional advantages (Jach & Malm, 2022). This yeast was also shown to be part of the normal human gut microbiota (Gouba & Drancourt, 2015).

Industrial good manufacturing practices production platforms based on engineered *Y. lipolytica* have been developed since the 2000s and include new white biotechnological applications, for example as a functional feed additive for animals or a source of human milk oligosaccharide for infant milk formulations (Guardiola et al., 2021; Madzak, 2021a). *Y. lipolytica* biomass from a proprietary wild‐type strain is commercialized as fodder yeast for farm and pet animals by Skotan SA (Chorzów, Poland), a company that also develops prebiotic/probiotic applications for this yeast (Jach & Malm, 2022; Madzak, 2021a; Zinjarde, 2014). Indeed, several in vivo studies reported probiotic properties of this yeast in mammals, birds, fish, crustaceans and molluscs (Guardiola et al., 2021). Beneficial health effects of *Y. lipolytica* on animals include productive performance, immune enhancement, redox balance and disease resistance. This yeast also improved fatty acid composition in muscle or fillet, haematobiochemical parameters, maturation and microbial populations of the gastrointestinal tract (Guardiola et al., 2021).

Since several decades, *Y. lipolytica* has been engineered to produce human compatible therapeutic proteins such as blood coagulation factor XIIIa, epidermal growth factor and single‐chain antibodies (Darvishi et al., 2019; Espejo‐Mojica et al., 2015; Gasmi et al., 2011; Hamsa et al., 1998; Madzak, 2021a; Madzak & Beckerich, 2013; Swennen et al., 2002; Tharaud et al., 1992; Tiels et al., 2012). Several *Y. lipolytica* strains have been engineered through deletion of their yeast‐specific mannosyltransferases and over and/or heterologous expression of other enzymes of the glycosylation pathways, to produce more human‐compatible glycoproteins (De Pourcq, Tiels, et al., 2012; De Pourcq, Vervecken, et al., 2012). Some recombinant enzymes produced by *Y. lipolytica* strains constitute the basis of different enzyme replacement therapies that are now marketed or on the edge of being marketed (Madzak, 2021b).

However, despite the huge attention that is being given to *Y. lipolytica* for the industrial manufacturing of various heterologous proteins (Madzak, 2021b; Madzak & Beckerich, 2013) and for probiotic effects, its potential for oral delivery of recombinant therapeutic proteins (particularly for their effect in the gastrointestinal tract, GIT) has seldom been evaluated in mammals and rarely in other vertebrates. Inspired by its use as feed and/or dietary supplement in pisci/aquaculture, several research teams have used *Y. lipolytica* as a vector for heterologous immunogenic proteins or antibacterial peptides to protect fish or shrimps against infections (Guardiola et al., 2021). In contrast, such studies are rarer in mammals but one unique study showed that freeze‐dried viral capsid proteins produced by *Y. lipolytica* can be used as an oral vaccine to protect mice against viral nervous necrosis (Luu et al., 2017).

In contrast to most other yeasts, *Y. lipolytica* secretes proteins predominantly via a cotranslational translocation pathway based on an efficient secretion signal recognition system similar to that of higher eukaryotes such as mammals (Swennen & Beckerich, 2007). Such pathway is particularly appropriate for the production of heterologous therapeutic proteins, which are often large complex proteins that have a mammalian/human origin. This yeast also compares favourably with other high secretor yeasts such as *Pichia pastoris* in terms of good product yield, even for large and complex proteins, and of reduced overglycosylation (Madzak, 2015, 2021a). These specific advantages constitute valuable assets in the potential development of *Y. lipolytica* as an oral delivery vector for the secretion of active therapeutic proteins in situ in the gut (Kim et al., 2015). As a proof of concept, we initially engineered two *Y. lipolytica* strains, the 1E07 wild‐type dairy strain and a laboratory strain from the Po1 series (Po1h, derived from the environmental W29 wild‐type strain – Madzak, 2021a), to produce the model fluorescent protein mCherry. The viability and persistence in vivo of these two live strains, as well as their ability to produce mCherry in the mouse intestine, was studied following oral administration. A comparison was then made with the viability and persistence of these two strains with *Lactobacillus plantarum* NCIMB8826 which is the “gold standard” *Lactobacillus* strain for studies of persistence in the GIT.

## RESULTS

### Development of constructs of mCherry producing *Y. lipolytica* 1E07 and Po1h strains

We constructed four recombinant *Y. lipolytica* strains able to produce the model fluorescent protein mCherry, starting from the 1E07 dairy strain or the Po1h laboratory strain. We used a previously validated *Y. lipolytica* integrative expression system, namely a vector from the series of widely used zeta‐based auto‐cloning expression vectors from which an expression/selection cassette devoid of bacterial sequences can be isolated and used to transform yeast cells (Madzak, 2015). The stability of constructions issued from such vectors has previously been demonstrated for more than 100 generations (Madzak, 2018; Madzak & Beckerich, 2013). Two different constructs were derived from each of these parental strains: (i) one carrying the *mCherry* coding sequence alone and (ii) the other one with a multiple cloning site (MCS) after the *mCherry* coding sequence, in order to allow future insertions of sequences encoding proteins of interest. The MCS includes eight restriction sites, all unique in the expression vector, to allow in future projects the insertion of coding sequences of interest for production of the corresponding protein in fusion with mCherry (Figures S1 and S2). The four newly constructed *Y. lipolytica* strains were named Yl1E07‐mCherryMCS, Yl1E07‐mCherry, YlPo1‐mCherryMCS and YlPo1‐mCherry. All the previously published or newly designed plasmids used for the construction of *L. plantarum* and *Y. lipolytica* strains from this work are described in Table 1. The genealogy of all the *L. plantarum* and *Y. lipolytica* strains used in this work is presented in Table 2.

**TABLE 1:** Plasmids used for strain construction.

Plasmid names	Description (relevant characteristics)	Reference or source
For *L. plantarum* strains construction:		
NICE® pNZ8148	Cm^r^, *L. lactis* pSH71 replicon	MoBiTec
pMEC275	pNZ8148 carrying mCherry cDNA optimized for *L. plantarum* codon bias fused to the *L. plantarum* P*ldh* promoter	Salomé‐Desnoulez et al., 2021
For *Y. lipolytica* strains constructions:		
pINA1317‐YlCWP110	Auto‐cloning vector for expression, secretion and/or surface display of heterologous proteins in *Y. lipolytica*, directed by the recombinant hp4d promoter. *Not*I restriction digest releases a *URA3*‐bearing expression/selection cassette devoid of bacterial sequences which is used to transform yeast cells.	Yue et al., 2008
pINA1317‐mCherry	pINA1317‐YlCWP110 bearing mCherry cDNA sequence (GenBank AY678264)	This work
pINA1317‐mCherryMCS	pINA1317‐YlCWP110 bearing mCherry cDNA sequence fused to a downstream Multiple Cloning Site region for possible ulterior insertion of an ORF of interest.	This work

Abbreviations: cDNA, complementary DNA; Cm^r^, resistance to chloramphenicol; ORF, Open Reading Frame.

**TABLE 2:** Yeast and bacterial strains used.

Strain names	Description (relevant characteristics)	Reference and/or source
*Lactobacillus plantarum* strains:		
NCIMB8826	Wild‐type strain isolated from human saliva	NCIMB^a^
Lp‐mCherry	Derivative of NCIMB8826 carrying the pMEC275 plasmid. Producer of mCherry fluorescent protein	Salomé‐Desnoulez et al., 2021
*Yarrowia lipolytica* strains:		
Po1h (CLIB 882)	Genetically modified laboratory strain, from the Po1 series of derivatives of W29 wild‐type strain. Auxotroph for uracil, deleted for extracellular proteases. Parent strain of fluorescent constructs	Madzak, 2021a CIRM‐Levures^b^
YlPo1‐mCherry	Po1h transformed by integration of a pINA1317‐mCherry vector expression/selection cassette. Prototroph, deleted for extracellular proteases, producer of mCherry red fluorescent protein	This work
YlPo1‐mCherryMCS	Po1h transformed by integration of a pINA1317‐mCherryMCS vector expression/selection cassette. Prototroph, deleted for extracellular proteases, producer of mCherry red fluorescent protein	This work
Po1t (CLIB 883)	Genetically modified laboratory strain, from the Po1 series of derivatives of W29 wild‐type strain. Prototroph, deleted for extracellular proteases. Same genetic background as Po1h. Used as non‐productive control	Madzak, 2021a CIRM‐Levures
1E07	Wild‐type strain isolated from Livarot French cheese. Parent strain of fluorescent constructs, also used as non‐productive control	Hébert et al., 2013 SayFood^c^
1E07‐ura	Derivative of 1E07 carrying a deletion of URA3 ORF obtained by the pop in / pop out method. Auxotroph for uracil	SayFood (unpublished work)
Yl1E07‐mCherry	1E07‐ura transformed by integration of a pINA1317‐mCherry vector expression/selection cassette. Prototroph, producer of mCherry red fluorescent protein	This work
Yl1E07‐mCherryMCS	1E07‐ura transformed by integration of a pINA1317‐mCherryMCS vector expression/selection cassette. Prototroph, producer of mCherry red fluorescent protein	This work

^a^ National Collection of Industrial, Food and Marine Bacteria (UK, https://www.ncimb.com/).

^b^ Centre International de Ressources Microbiennes‐Levures (INRAE yeast collection, France, https://www6.inrae.fr/cirm_eng/Yeasts).

^c^ SayFood laboratory private strain collection.

**FIGURE 1:**
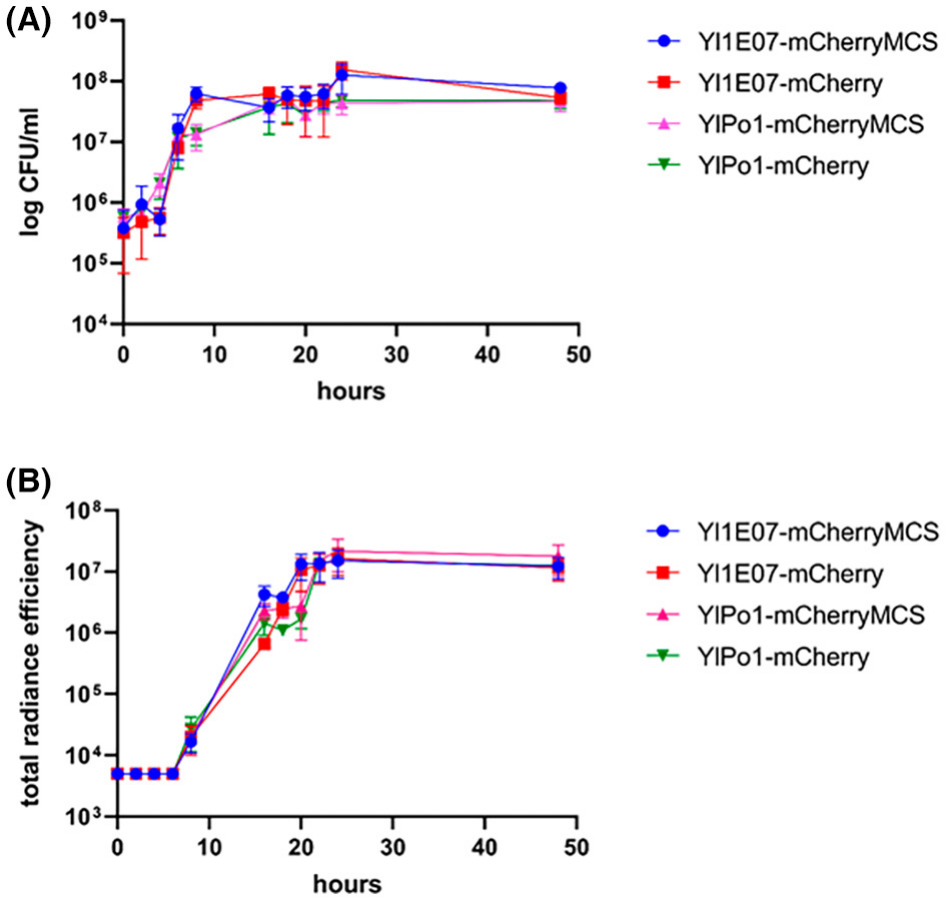
In vitro growth and fluorescence kinetics of Yl1E07‐mCherryMCS, Yl1E07‐mCherry, YlPo1‐mCherryMCS and YlPo1‐mCherry strains. (A) Growth curves of engineered *Y. lipolytica* strains grown in modified YPD medium at 30°C. The values shown are the means from three independent cultures. The error bars correspond to the standard deviation. (B) Fluorescence signal kinetics of the engineered strains. Strain culture samples were adjusted to a similar yeast concentration and aliquoted into black microplates. The values shown are the means from two independent cultures, measured in triplicate. Results are given as the total radiance efficiency (in [(p/s/cm^2^/sr)/(μW/cm^2^)]). The background fluorescence for 1E07 and Po1t non‐productive control strains has been subtracted from each corresponding value. The error bars correspond to the standard deviation.

We observed that the growth curves of the four fluorescent *Y. lipolytica* recombinant strains were similar (Figure 1A) and that the production of mCherry in these strains did not affect their growth in comparison to the non‐producing controls, 1E07 and Po1t, respectively (data not shown). The fluorescence signals produced in vitro by Yl1E07‐mCherryMCS, Yl1E07‐mCherry, YlPo1‐mCherryMCS and YlPo1‐mCherry were quantified in aliquots collected at different times during yeast cultivation in liquid medium and were shown to be similar in fluorescence levels and kinetics (Figure 1B). These results demonstrate that, despite their different genetic backgrounds, the two parental strains exhibited a similar potential for active mCherry production and that the insertion of a MCS is not detrimental to the construction efficiency. In consequence, we chose to focus only on the two Yl1E07‐mCherry and YlPo1‐mCherry strains for the following experiments.

### The two fluorescent *Y. lipolytica* strains persist similarly to mCherry producing *L. plantarum* NCIMB 8826 in the mouse intestine after oral administration

In mice, oral administration of high doses of either Yl1E07‐mCherry and or YlPo1‐mCherry (8 × 10^9^ CFU/day) for five consecutive days did not show any apparent detrimental effect on mouse physical activity, weight nor any sign of colon inflammation (Table 3).

**TABLE 3:** Effects of intragastric administration ofYl1E07‐mCherry and YlPo1‐mCherry in mice.

Buffer or strain	No. of mice	Physical activity score^a^	% variation in body weight (day 7‐day 1)	Colon inflammation^b^
NaHCO_3_ ^c^	10	1 ± 0	1.65 ± 0.5	No
Yl1E07‐mCherry	10	1 ± 0	0.7 ± 0.7	No
YlPo1‐mCherry	10	1 ± 0	0.8 ± 0.65	No

*Note*: Values are means ± standard errors.

Abbreviation: ND, not determined.

^a^ The physical activity of mice was recorded independently by two investigators who were blinded to treatment before sampling. They used a predefined scoring system ranging from 1 (healthy and very active) to 5 (agony and lethargic). This scoring system as already been described (van Griensven et al., 2002) and is based on rating physical activity and food intake using spontaneous activity of the mice, and spontaneous food intake.

^b^ After dissection at day 5, two independent observers blindly scored the macroscopic inflammation of the colon by using the Wallace score (Wallace et al., 1989). The Wallace score rates macroscopic lesions on a scale from 0 to 8 based on features reflecting inflammation, such as hyperemia (score 1 to 2), moderate to intense thickening of the bowel (score 2 to 3) and the gradual extent of 1 cm‐long ulceration (score 3 to 8), according to the maximal mouse colon length.

^c^ Administration of NaHCO3 buffer is used as a negative control.

**FIGURE 2:**
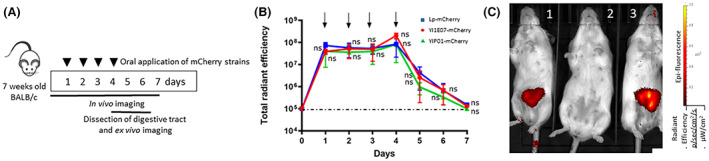
Persistence of mCherry producing *Y. lipolytica* and *L. plantarum* strains and after four daily oral administrations in mice. (A) The experimental design. Four groups of mice were constituted: (i) mice given Lp‐mCherry for 4 days (8 × 10^9^ CFU), (ii) mice given Yl1E07‐mCherry for 4 days (8 × 10^9^ CFU), (iii) mice given YlPo1‐mCherry for 4 days (8 × 10^9^ CFU) and (iv) negative representative control healthy mice given non‐fluorescent wild‐type *Y lipolytica* 1E07. Whole body imaging was performed from day 1 to day 7. Mice were euthanized on days 4, 5 and 6, and the GITs were excised for ex vivo imaging. This whole experiment was repeated three times (as three independent experiments). (B) From day 0 to 7, the total radiant efficiency in [(p/s/cm^2^/sr)/(μW/cm^2^)] in whole animals for each set of 6 mice is shown, with its standard deviation. The background fluorescence is represented by a dashed line. Intergroup differences were assessed using the Kruskal–Wallis non‐parametric test and no statistical difference (ns) was observed between the Lp‐mCherry and Yl1E07‐mCherry/Yl1Po1‐mCherry fed groups (with *p* > 0.05 for each time point). Fluorescent background levels obtained from healthy mice given non‐fluorescent wild‐type *Y lipolytica* 1E07, *Y lipolytica* Po1 or *L. plantarum* strains (used as negative controls) were similar (data not shown). (C) A representative image of three mice: (1) one mouse administered with Yl1E07‐mCherry on day 4, (2) a representative negative control mouse, representing the background fluorescence, and (3) one mouse administered with Yl1Po1‐mCherry on day 4. Fluorescence emission between 575 and 650 nm was measured with a high‐resolution filter after excitation at 460, 500 and 535 nm, resulting in the acquisition of several images. Live imaging software was then used to subtract the background fluorescence by spectral unmixing. The colour bar indicates the radiant efficiency [(p/s/cm^2^/sr)/(μW/cm^2^)].

The fluorescence signals emitted by both Yl1E07‐mCherry and YlPo1‐mCherry were quantified and compared with the mCherry producing *L. plantarum* NCIMB 8826 (Lp‐mCherry) strain in live mice, after four daily oral administrations of 8 × 10^9^ CFU dose of each strain. The results obtained using the IVIS® Lumina imaging system with three independent experiments are shown in Figure 2. The strains' respective fluorescence signals were quantified daily by direct imaging in six anaesthetised mice per group. The signals from both fluorescent *Y. lipolytica* and Lp‐mCherry groups were strong and did not differ significantly between day 1 and the last day of oral administration (day 4). The in vivo signals from both *Y. lipolytica* and *L. plantarum* fluorescent strains fell to background levels by day 7 (Figure 2B).

The persistence of the fluorescent strains (*Y. lipolytica* and *L. plantarum*) and their respective fluorescent signals were also investigated in the faeces of mice (Figure S3). For these experiments, we focused on the comparison of Yl1E07‐mCherry with Lp‐mCherry. Both strains persisted for a long time, respectively for 8 and 7 days (day 12 and day 11), after the last inoculation (day 4) and maintained themselves at levels ranging from 5 × 10^6^ to 10^2^ CFU/100 mg of faeces from days 5 to 12 and days 5 to 9, respectively. Lp‐mCherry fluorescent signal was detected in faeces 1 day after the last dose (given at day 4) while the Yl1E07‐mCherry signal was detected at lower levels and was undetectable at day 5. The fluorescent system allowed the detection of mean bacterial quantities in faeces as low as 10^6^ CFU/100 mg of faeces and 10^7^ CFU/100 mg faeces for Yl1E07‐mCherry.

### Characterization of the behaviour of both fluorescent *Y. lipolytica* strains in the gut

**FIGURE 3:**
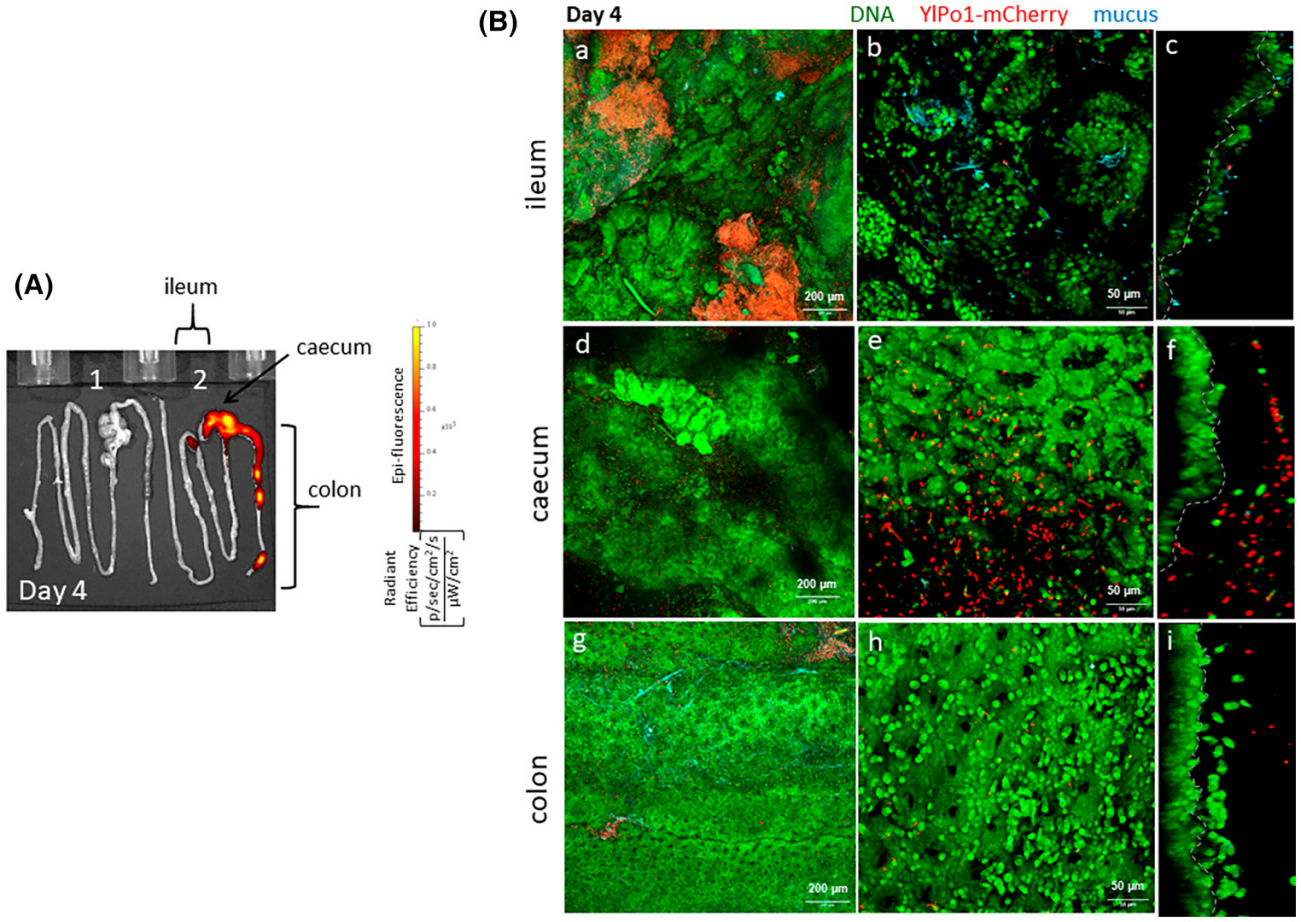
Localization of YlPo1‐mCherry strains in freshly collected digestive tracts using ex vivo fluorescence and confocal microscopy imaging on day 4 (i.e., after four daily oral administrations). Healthy mice received a daily dose of 7 × 10^9^ CFU of YlPo1‐mCherry for 4 consecutive days via the intragastric route as described previously. Intestines (n = 4) were imaged ex vivo and stained with acriflavine (staining the nucleus in green) and wheat germ agglutinin Alexa Fluor 633 (in blue). (A) Six mice were sacrificed each day and one representative image of two mouse digestive tracts is shown, at day 4, from the GIT of mice fed with *Y. lipolytica* Po1t non‐fluorescent negative control (1), or YlPo1‐mCherry (2). The colour bar indicates the radiant efficiency [(p/s/cm^2^/sr)/(μW/cm^2^)]. (B), a to i: Confocal microscopy imaging, with representative 10× (a, d and g, scale bar: 200 μm) and 25× (b, e and h, scale bar: 50 μm) tiling images of the mouse ileum (a, b), caecum (d, e) or colon (g and h). A representative 3D‐rendered image of the mouse ileum (c), caecum (f) and colon (i) is shown on the right. A boundary line between the intact tissue and the desquamated cells has been added to the orthogonal views to precisely delimit the border of the tissues and thus better visualize the yeasts respectively in the lumen of the intestine or in contact with host cells.

**FIGURE 4:**
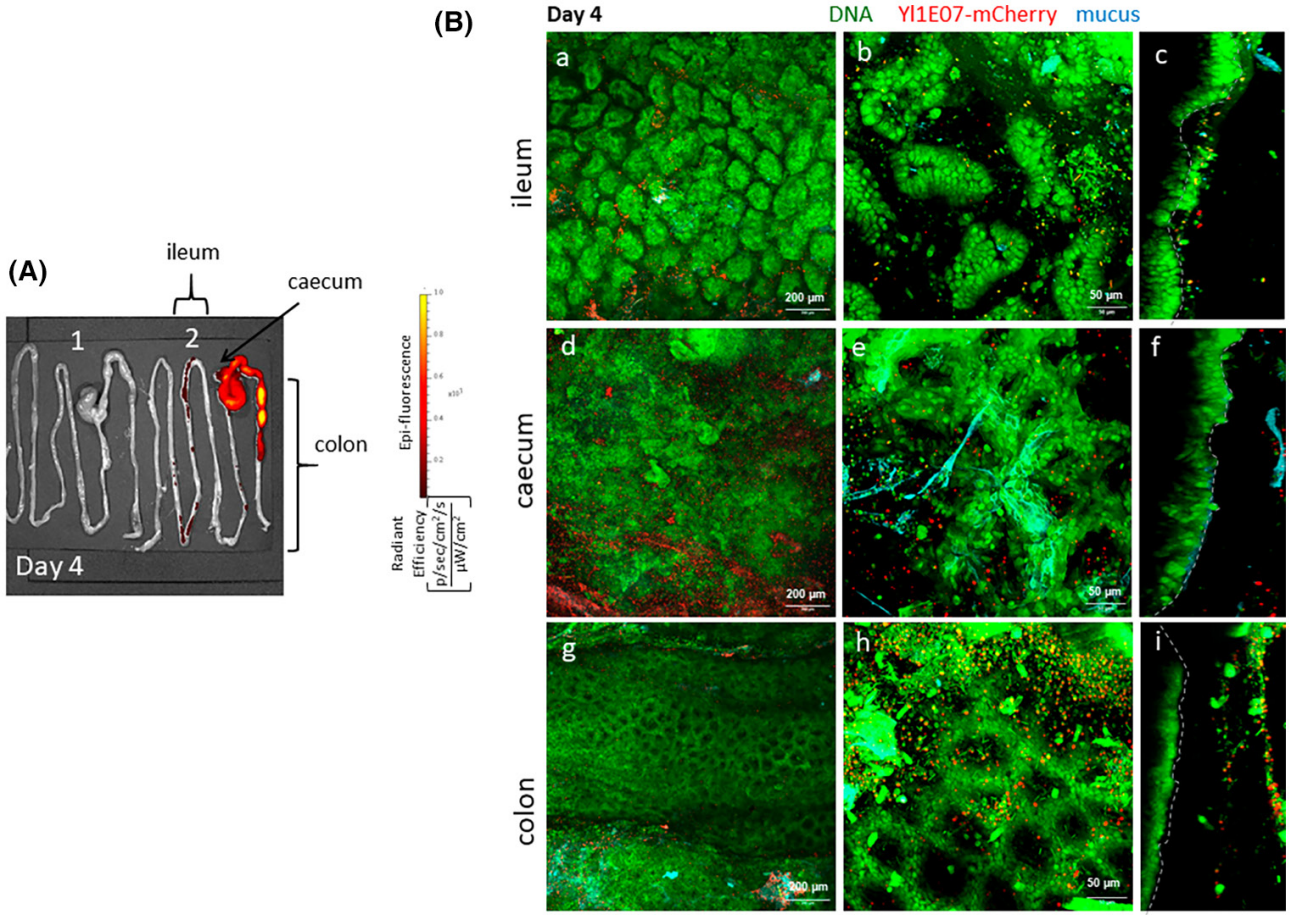
Localization of Yl1E07‐mCherry strains in freshly collected digestive tracts using ex vivo fluorescence and confocal microscopy imaging on day 4 (i.e., after four daily oral administrations). Healthy mice received a daily dose of 7 × 10^9^ CFU of Yl1E07‐mCherry for 4 consecutive days via the intragastric route as described previously. Intestines (*n* = 4) were imaged ex vivo and stained with acriflavine (staining the nucleus in green) and wheat germ agglutinin Alexa Fluor 633 (in blue). (A) Six mice were sacrificed each day and one representative image of two mouse digestive tracts is shown, at day 4, from the GIT of mice fed with *Y. lipolytica* 1E07 negative non‐fluorescent control (1), or Yl1E07‐mCherry (2). The colour bar indicates the radiant efficiency [(p/s/cm^2^/sr)/(μW/cm^2^)]. (B), a to i: Confocal microscopy imaging, with representative 10× (a, d and g, scale bar: 200 μm) and 25x (b, e and h, scale bar: 50 μm) tiling images of the mouse ileum (a, b), caecum (d, e) or colon (g and h). A representative 3D‐rendered image of the mouse ileum (c), caecum (f) and colon (i) is shown on the right. A boundary line between the intact tissue and the desquamated cells has been added to the orthogonal views to precisely delimit the border of the tissues and thus better visualize the yeasts respectively in the lumen of the intestine or in contact with host cells.

To extend our studies on the persistence of *Y. lipolytica* 1E07‐ and Po1h‐derived strains in the GIT, we next characterized both strains' behaviour in different intestinal compartments (ileum, caecum and colon) via ex vivo fluorescence and confocal microscopy of freshly collected mouse tissues after four daily oral administrations (Figure 3 for YlPo1‐mCherry and Figure 4 for Yl1E07‐mCherry). Tissues for whole body imaging were collected from the same mice after dissection. The ileum, colon and caecum were opened, stained and observed by confocal microscopy within 30 minutes of removal without any fixation. This method enabled us to visualize the thickness of intestinal compartments using deep imaging (z plane) and large expanses (x‐y planes) of the mucosa were captured in full z depth by tiling the acquired images.

From day 1 to day 4 (the last day of oral administration of the strain), the ex vivo fluorescence signals were strong for both strains which were localized predominantly in the caecum and colon with lower signals in the ileum as well (data not shown for day 1 to day 3). We also showed that large numbers of both yeast strains were present on large expanses of the mucosa of ileum, caecum and colon (10x objective; Figures 3B and 4B and 25x objective; Figures 3B and 4B) using confocal microscopy images. The zoomed three‐dimensional (3D)‐rendered images were prepared by thresholding the signals from the cell nuclei, mucus and bacteria and creating an iso‐surface to better visualize the respective yeasts in contact with host cells. The extended orthogonal view projections of ileum, caecum and colon enabled us to confirm that both yeast strains (in red) were essentially found in the lumen and above and inside the crypts. Both *Y. lipolytica* strains were found close to endogenous bacteria from the gut microbiota (cocci and bacilli, represented by green spots in the lumen) but rarely directly in contact with intestinal cells (Figures 3 and 4). In some representative images, both strains were found close to senescent cells which were being eliminated during epithelial renewal (Skoczek et al., 2014). Moreover, both strains were found both in and outside the mucus layer.

**FIGURE 5:**
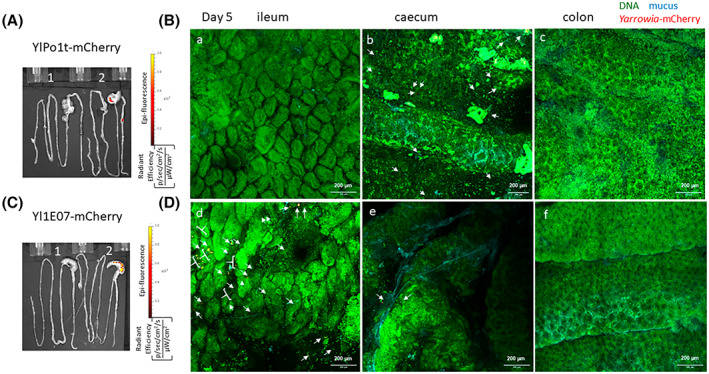
Detection and observation of *Y. lipolytica* strains in the GIT on day 5 in freshly collected digestive tracts using ex vivo fluorescence and confocal microscopy imaging (after four daily oral administrations). (A, C) One representative image of two mouse digestive tracts is shown, from the GIT of mice fed with respectively, for (a), *Y. lipolytica* Po1t negative control (1), or YlPo1‐mCherry (2) and, for (C), *Y. lipolytica* 1E07 negative control (1), or Yl1E07‐mCherry (2). The colour bar indicates the radiant efficiency [(p/s/cm^2^/sr)/(μW/cm^2^)]. (B, D) Confocal microscopy imaging, with representative 10× (scale bar: 200 μm) tiling images of the mouse ileum (a, d), caecum (b, e) or colon (c and f). White arrows and brackets indicate the few persistent red mCherry producing *Yarrowia* strains.

On day 5, the three strains were also localized predominantly in the ileum, caecum and colon, with only very low ex vivo remaining signals in the whole intestines (Figure 5A). These results are in line with our whole‐body imaging results with live mice (Figure 2) and confirmed by our confocal microscopy images (Figure 5B). Indeed, only very few individual red *Y. lipolytica* cells were still present in the ileum, colon and caecum. On day 6, no individual red cells (both strains) could be detected in the different intestinal compartments (data not shown).

## DISCUSSION

In this study, we developed novel fluorescently labelled *Y. lipolytica* strains to study their persistence in the mouse gastrointestinal tract as well as their interactions with the host.

For the first time, we showed that recombinant *Y. lipolytica* strains were able to produce a heterologous protein, the fluorescent mCherry, directly in the mouse GIT. To the best of our knowledge, this is the first report of *Y. lipolytica* heterologous protein production in situ in the intestine. Previous studies involving the production of mCherry (or other fluorescent proteins such as GFP – Yue et al., 2008) in *Y. lipolytica* were limited to in vitro applications such as the production and purification of heterologous proteins (Bulani et al., 2012) and the design of functional oleosomes or biosensors (Han et al., 2013; Wei et al., 2020). We have also demonstrated that the promoter hp4d used in our constructs was active in the intestine. This promoter, a recombinant structure composed of four direct repeats of the UAS1B sequence from the *Y. lipolytica XPR2* promoter upstream from a minimal *LEU2* promoter of this yeast (Madzak et al., 2000), enables a strong quasi‐constitutive expression and since decades constitutes a preferred tool for heterologous expression in *Y. lipolytica* (Madzak, 2015, 2018). Expression driven by hp4d is as strong as that from the best native promoters known and, in addition, remarkably independent from environmental conditions (pH, carbon and nitrogen sources – Madzak et al., 2000). Moreover, it develops its full strength mostly at the beginning of the stationary phase of cell growth, which naturally separates a growth phase from an expression phase without the need of any induction step, a situation particularly beneficial to efficient heterologous protein production (Madzak, 2015, 2018) that prompted its selection for this work.

No significant difference was observed between the results obtained with the fluorescent *Y. lipolytica* strains bearing the *mCherry* sequence alone or followed by the MCS, in any of the experiments in which they were compared. Thus, the presence of the MCS as an additional amino acid sequence fused to mCherry does not interfere with the emitted fluorescence. This demonstration paves the way for possible future uses of this system for producing fusion proteins in the GIT.

In order to maintain the number of animals used in this study as low as possible for ethical reasons, only two different *Y. lipolytica* strains have been tested and used to express *mCherry*, (i) a laboratory strain, Po1h, derived from the environmental W29 strain (isolated from Paris sewers – Madzak, 2021a) and (ii) a dairy strain, 1E07. The Po1h laboratory strain belongs to the Po1 series of recipient strains that are the most widely used worldwide as *Y. lipolytica* engineering tools (Madzak, 2015, 2018; Madzak & Beckerich, 2013). The dairy 1E07 strain was chosen as an example of the many alimentary *Y. lipolytica* strains that earned this yeast a GRAS status. The similitude of the results obtained with the two kinds of fluorescent strains, namely YlPo1‐mCherryMCS and YlPo1‐mCherry on the one hand and Yl1E07‐mCherryMCS and Yl1E07‐mCherry on the other, is interesting because these two categories do not differ only by their different natural genetic backgrounds, but also by the fact that the Po1h‐derived strains are unable to secrete proteases. Namely, both *Y. lipolytica* extracellular proteases (AEP, encoded by *XPR2* gene, and AXP) have been deleted in the Po1 series of strains. The fact that the mCherry fluorescence levels observed with recombinant strains derived from either the dairy 1E07 strain or the ΔAEP ΔAXP Po1h strain are similar indicates that the proteases that could be secreted by Yl1E07‐mCherryMCS and Yl1E07‐mCherry did not significantly contribute to degrade the mCherry protein produced in situ in the GIT, as expected for a protein that was not released into the cell environment.

Even if lactic acid bacteria have till now been the main candidate hosts for the delivery of eukaryotic therapeutic proteins in the gut (Daniel et al., 2011; Plavec & Berlec, 2019; Rosales‐Mendoza et al., 2016), yeasts can be more advantageous, especially when a eukaryotic environment is required for the functional expression of heterologous genes. Moreover, this makes it possible to ensure the absence of bacterial sequences liable to promote gene transfer to host bacteria as the heterologous gene is integrated directly in the yeast's genome in comparison with the use of exogenous plasmids with lactic acid bacteria. Indeed, *Saccharomyces cerevisiae* and *Saccharomyces boulardii* have already been used as safe and robust vehicles for efficient oral delivery of either active antigens or drugs in the gut with the final goal to prevent and treat intestinal pathologies in preclinical studies (Blanquet et al., 2001; Garrait et al., 2007, 2009; Heavey & Anselmo, 2021; Kenngott et al., 2016; Liu et al., 2020; Rosales‐Mendoza et al., 2016). However, there are some limitations to the use of *S. cerevisiae* to express efficiently some complex heterologous proteins, such as most of those that can be used as therapeutic proteins. These drawbacks, which include notably low yields (especially for secreted proteins) and a tendency to hyperglycosylate some proteins (leading to activity and/or clearance problems), has prompted the search for alternative yeast systems (Baghban et al., 2019). Among the non‐conventional yeasts currently used for biotechnological applications, *Y. lipolytica* is one of the preferred choices, and the Po1 series of recombinant strains is especially successful in recombinant protein production (Baghban et al., 2019; Madzak, 2021a).

In our study, we have shown that *Y. lipolytica* fulfils the qualifications for such vehicles in a unique manner: this yeast transiently persists at least 1 week after four daily oral administrations (as determined by faecal examination) and maintains the active expression of mCherry in the GIT. In addition, *Y. lipolytica* secretes proteins predominantly via a cotranslational translocation pathway based on an efficient secretion signal recognition system similar to that of higher eukaryotes, in contrast to other yeasts (Swennen & Beckerich, 2007). The GRAS status of *Y. lipolytica* makes it both environmentally friendly and attractive for use as an oral delivery vector for the secretion of active therapeutic proteins in the gut (Markham & Alper, 2018). Moreover, among the large range of genetic engineering tools available in *Y. lipolytica*, there are many that are used such as the auto‐cloning vector system (insertion of an expression/selection cassette) used in this work or the more recently developed CRISPR tools that allow avoiding the presence of any bacterial DNA (and notably antibiotic resistance genes) in the recombinant strains (Madzak, 2021a, b). The genetically modified microorganisms generated using such strategies can preserve the GRAS status of *Y. lipolytica* and eliminate all risk of possible horizontal gene transfer (notably of antibiotic resistance) to the gut bacteria.

We have clearly shown that both Yl1E07‐mCherry and YlPo1‐mCherry strains produce mCherry in the mouse GIT, especially in the caecum and colon. Genetically engineered *S. cerevisiae* and *S. boulardii* have been shown to produce different model proteins directly in the mouse or rat digestive tracts, especially in the large intestine (Garrait et al., 2007, 2009; Heavey & Anselmo, 2021; Liu et al., 2020) but their behaviour in the gut was not studied extensively. We also showed that both externally administered *Y. lipolytica* strains persist like *L. plantarum* NCIMB8826 and do not replicate actively and do not permanently colonize the GIT. Similar findings have been demonstrated in various mouse experimental studies with *Saccharomyces* strains (*S. cerevisiae* and *S. boulardii*) showing that orally administered yeasts survive the harsh condition of the GIT but do not colonize it (Cordonnier et al., 2015; Liu et al., 2020). Moreover, two studies in humans showed that the daily ingestion of 10^8^ cells of *S. cerevisiae* or *S. boulardii* for 5 days results in a maximum faecal count of 10^5^ CFU/g and that both yeast levels decrease in faeces once the administration has stopped (Klein et al., 1993; Pecquet et al., 1991).

Caecum and colon were found to be the predominant sites for persistent *Y. lipolytica* cells in mice by ex vivo imaging as already demonstrated with *L. plantarum* NCIMB8826 (Daniel et al., 2013; Salomé‐Desnoulez et al., 2021). We then used confocal microscopy of freshly collected intestinal tissues to image both *Yarrowia* strains on the single‐cell state. Individual *Yarrowia* cells were successfully visualized while transiting through the ileum, caecum and colon of mice. *Yarrowia* cells persisted in the intestine for 7 days after the last oral administration. To the best of our knowledge, this is the first report of imaging orally administered individual yeasts in the mouse gut since novel tools and strategies have been developed to study the spatial organization of gut bacteria (Earle et al., 2015; Geva‐Zatorsky et al., 2015; Hudak et al., 2017; Whitaker et al., 2017). Moreover, extended orthogonal 3D projections enabled us to visualize individual yeasts essentially in the lumen (in contact with gut bacteria and mucus) and were rarely seen being in contact with epithelial cells. The absence of commensal or orally administered bacteria in direct contact with gut epithelial cells was already reported in the literature (Hudak et al., 2017; Swidsinski et al., 2007) and this can be explained by the presence of the mucus barrier with a varying thickness throughout the small intestine and colon (Paone & Cani, 2020). In comparison with gut bacteria, less data are available concerning the fungal microbiota of human and mice because of their low level of presence (0.1% of total microorganisms) (Li et al., 2018). However, recent studies have shown that fungi, although present in small numbers in the GIT and with a lower diversity than bacteria, are essential for the homoeostasis of the entire gut microbiota and interaction with intestinal immunity (Wu et al., 2021).

Interestingly, Bazan et al. (2018) showed that a *Y. lipolytica* strain incubated in vitro with monocyte‐derived dendritic cells from healthy donors induced their maturation and the secretion of inflammatory cytokines as well as T cell markers. This suggests that *Y. lipolytica* may serve as an immunostimulatory yeast and can be used as a delivery vehicle of vaccines, because T cell responses critically depend on the appropriate antigen presenting cell activation. Taken as a whole, our results have shown that safe fluorescent *Y. lipolytica* strains constitute novel tools to study the persistence and dynamics of orally administered yeasts which could be used in the long term as oral delivery vectors for the secretion of active therapeutic proteins in the gut.

## EXPERIMENTAL PROCEDURES

### Recombinant strains and growth conditions

Yeast, bacterial strains and plasmids used for strain constructions are listed in Tables 1 and 2. The construction of the Lp‐mCherry strain was described previously (Salomé‐Desnoulez et al., 2021). Briefly, the *L. plantarum* codon‐optimized *mCherry* gene under the control of P*ldh* was inserted into the expression vector NICE® pNZ8148 and the resulting plasmid was introduced into *L. plantarum* NCIMB8826 by electrotransformation (Salomé‐Desnoulez et al., 2021). *L. plantarum* strains were grown at 37°C in MRS medium (Difco, Becton Dickinson). Chloramphenicol (Sigma‐Aldrich) was added to culture media for bacterial selection when necessary, at a final concentration of 10 μg/ml. All recombinant *Y. lipolytica* strains used in this study were derived from either the previously described Po1h laboratory strain (*MatA*, *ura3‐302*, *xpr2‐322*, *axp1‐2*; Ura^−^, Suc^+^, ΔAEP, ΔAXP), a commonly used *Y. lipolytica* recipient strain (Madzak, 2015, 2018; Madzak & Beckerich, 2013), or from the wild‐type 1E07 dairy strain (Hébert et al., 2013). An Ura^−^ derivative of 1E07 (1E07‐ura) was obtained using the classical pop‐in/pop‐out method (Barth & Gaillardin, 1997) to generate a *URA3* ORF deletion. Two different *URA3*‐bearing expression/selection cassettes carrying the *mCherry* ORF gene (GeneBank accession number HM771696) were used to transform both uracil defective strains, Po1h and 1E07‐ura, in order to generate the different fluorescent recombinant *Y. lipolytica* strains, using the classical LiAc and heat shock method (Madzak et al., 2000). These expression/selection cassettes were generated by a *Not*I restriction digest of the DNA from either pINA1317‐mCherry or pINA1317‐mCherryMCS auto‐cloning expression vectors. These two vectors were constructed by inserting respectively a *Sfi*I‐*Bgl*II fragment (carrying mCherry only) or a *Sfi*I‐*Bam*HI fragment (carrying mCherry fused to the MCS) from the “mCherry‐MCS” DNA sequence (cf. Figure S1), synthetized by Eurogentec France (Angers), between the *Sfi*I and *Bam*HI unique restriction sites (*Bgl*II and *Bam*HI having ligation‐compatible overhangs) of the previously described pINA1317‐YlCWP110 auto‐cloning expression vector (Yue et al., 2008) (Figure S2). These insertions put the *mCherry* ORF (+/− MCS) downstream from the strong quasi‐constitutive hp4d promoter used to direct its expression in *Y. lipolytica*. Transformants obtained by integration of each *URA3*‐bearing expression/selection cassette carrying the *mCherry* ORF, in the genome of one or the other *Y. lipolytica* strain, were selected on minimal medium (YNB N_5000_ – Madzak et al., 2000) (Figure S2). For each construction (Yl1E07‐mCherry, Yl1E07‐mCherryMCS, YlPo1‐mCherry and YlPo1‐mCherryMCS), a dozen of independent transformants were checked visually for fluorescence level using an Olympus BX51 microscope and the most fluorescent respective transformant was selected for further analysis. All *Y. lipolytica* strains were grown in modified YPD medium (1% yeast extract, 2% dextrose and 2% tryptone) at 30°C in shake‐flasks at 200 rpm.

### In vitro fluorescence measurement

The fluorescence of the various recombinant strains was quantified as described previously (Salomé‐Desnoulez et al., 2021) (Figure S4). Briefly, the strains were grown to different time points, harvested by centrifugation and washed with phosphate buffered saline (PBS). Fifty microlitres of each culture, adjusted to approximately 3.5 × 10^10^ CFU/ml, was distributed in black microplates (Nunc, Thermo Fisher) and fluorescence was measured at room temperature on the IVIS Lumina XR imaging system (Perkin Elmer) using the Living Image software (Perkin Elmer). Strains were compared on the basis of total radiance efficiency [(p/s/cm^2^/sr)/(μW/cm^2^)]. Emission of fluorescence was measured with high resolution filters with different excitation wavelengths (460, 500 and 535 nm) and emission wavelengths (between 575 and 650 nm) resulting in acquisition of several images with an exposition time of 3 s (the use of the emission wavelengths was optimized to obtain the highest signals in vitro). The Living Image software was then used for generating spectrally unmixed images and quantification of each fluorescent signal after removal of the autofluorescence background. Each individual well was manually selected as a region of interest (ROI). *L. plantarum* NCIMB8826 containing the empty vector pNZ8148, *Y. lipolytica* 1E07 and *Y. lipolytica* Po1t were used to measure the background fluorescence as negative controls of the corresponding fluorescent strains.

### Animal experiments and ethics statements

BALB/c mice (female, 6 weeks of age) were purchased from Charles Rivers (L'Arbresle, France) and maintained in pathogen‐free animal holding facilities. The animal experiments were conducted in accordance with the relevant guidelines and regulations. Indeed, the animal experiments complied with French legislation (Government Act 87–848). All the studies were approved by the local investigation ethics review board (Nord‐Pas‐de‐Calais CEEA N°75, Lille, France) and French government (agreement n° APAFIS#201608251651940). Yeast and bacterial strains were grown to the stationary phase (16 h for *L. plantarum* and 24 h for *Y. lipolytica* strains), harvested by centrifugation and washed with PBS. Mice received various amounts of yeast or bacteria administered by oral gavage in 200 μl buffer (0.2 M NaHCO3 buffer containing 1% glucose, pH 8).

### In vivo persistence of strains in the GIT of mice and in vivo fluorescence imaging

Mice received a daily dose of 7 × 10^9^ CFU of live Lp‐mCherry, Yl1E07‐mCherry or YlPo1‐mCherry for 4 consecutive days via the intragastric route. This physiological dose was administered to mice in order to obtain the highest signals emitted by the different strains for comparison. Control mice received the corresponding *Y. lipolytica* non‐fluorescent negative control strain (1E07 or Po1t) in the different experiments.

Fluorescence imaging was performed daily from day 0 to day 7 using a multimodal IVIS Lumina XR imaging system (Perkin Elmer). Prior to imaging, mice were anaesthetised with 2% isoflurane. Two hours after the oral administration of the respective recombinant strains, all mice were placed into the specimen chamber of the IVIS imaging system where a controlled flow of 1.5% isoflurane in air was administered through a nose cone via a gas anaesthesia system (Tem Sega). Emission of fluorescence was measured with high resolution filters with different excitation wavelengths (500, 535, 570, 605 and 640 nm) and emission wavelengths (between 695 and 770 nm) resulting in acquisition of several images with an exposition time of 3 s (the use of the emission wavelengths was optimized to obtain the highest signals in whole body mouse imaging). The Living Image software was then used for generating spectrally unmixed images and quantifying each fluorescent signal. Fluorescence was quantified using the Living Image software (given as the radiance efficiency in photons s^−1^ cm^−2^ steradian^−1^ per μW cm^−2^ and p/s, respectively). For anatomical localization, a pseudocolour image representing detected light intensity was generated using the Living Image software and superimposed over the grey scale reference photography. For each individual mouse, there was only one ROI corresponding to the mouse digestive tract and this ROI was manually selected.

Faecal samples were collected individually at different time points and mechanically homogenized at 100 mg of faeces/ml in MRS medium for *L. plantarum* NCIMB8826 or modified PPB medium (dextrose 20 g, yeast extract 1.32 g, ammonium chloride 1.32 g, MgSO_4_ × 7H_2_O 0.24 g, CuSO_4_ 0.025 g and thiamine 0.033 mg per litre) supplemented with tetracycline (500 μg/mL) for the recombinant *Y. lipolytica* (optimized medium for detection of *Y. lipolytica* in mice faeces). Dilutions were plated on to the respective selective media described above and incubated before enumeration. No tetracycline resistant yeast and no chloramphenicol resistant bacterium were detected in non‐inoculated mice. Mice were sacrificed by cervical dislocation, and mouse digestive tracts (from end of stomach to rectum) were immediately excised for ex vivo imaging.

### Fluorescence microscopy: Sample preparation, confocal microscopy and image analysis

Mice received a daily dose of 7 × 10^9^ CFU of live Yl1E07‐mCherry or YlPo1‐mCherry for 4 consecutive days via the intragastric route and observed for confocal imaging at days 4, 5 and 6 as described previously (Salomé‐Desnoulez et al., 2021). Briefly, the mouse colon, caecum and ileum (each 1.5 cm long and at the same spot in each mouse) were opened longitudinally, stained with 1 mg/ml of acriflavine (Sigma‐Aldrich) as well as 5 μg/mL of wheat germ agglutinin Alexa Fluor 633 (Thermo Fisher) and then mounted in a Petri dish and immobilized in low melting agarose 1.5%. The samples are observed within 30 min. Imaging was performed using a Leica‐Sp8 upright confocal microscope equipped with a resonant scanner and hybrid detector (confocal TCS SP8, Leica microsystems). Stack of tiled images with 10% overlap between fields were acquired with the 10× objective (HCX PL FluoTAR 10x/0.30 PH1 objective, Leica microsystems) to obtain wide images of the tissues. A 25× high numerical aperture objective (HC FluoTar L 25x /0.95 W, Leica microsystems) was also used to acquire deep tissue image stacks. Representative images shown in Figures 3, 4, 5 were selected from a total of approximately 10 mice.

As described previously (Salomé‐Desnoulez et al., 2021), the image contrast was improved by deconvolution (Huygens, SVI Scientific Volume Imaging). In Figures 3 and 4, 10× and 25× image stacks were reduced to a two‐dimensional maximum intensity Z‐projection image (Fiji/ImageJ, https://imagej.nih.gov/). To localize the bacteria in the thickness of the stack, the extended orthogonal view projection and 3D montages were produced using Imaris9.3.1 software (Bitplane, Oxford Instruments). Extended orthogonal view projections over 50 μm in YZ and 3D montages were produced using Imaris 9.8.0 software (Bitplane, Oxford Instruments) to facilitate the visualization of the yeasts in the thickness of the stack.

### Statistical analysis

All analyses were performed by comparing experimental groups to their respective controls using the Kruskal Wallis non‐parametric test (GraphPad Prism 6.0). Data are presented as means ± SEM. Differences were judged to be statistically significant when the *p*‐value was <0.05.

## AUTHOR CONTRIBUTIONS

**catherine MADZAK:** Conceptualization (equal); data curation (equal); formal analysis (equal); funding acquisition (equal); investigation (equal); methodology (lead); project administration (supporting); resources (equal); supervision (equal); validation (equal); writing – original draft (equal); writing – review and editing (equal). **Sabine POIRET:** Conceptualization (equal); data curation (equal); formal analysis (equal); investigation (equal); methodology (equal); resources (equal); validation (equal); visualization (equal). **Sophie Salomé‐Desnoulez:** Conceptualization (equal); data curation (equal); formal analysis (equal); investigation (equal); methodology (equal); resources (equal); software (equal); validation (equal); visualization (equal). **Benoit Foligné:** Data curation (equal); funding acquisition (equal); investigation (equal); validation (equal); visualization (equal); writing – original draft (equal); writing – review and editing (equal). **Frank Lafont:** Funding acquisition (equal); investigation (equal); project administration (equal); writing – original draft (equal); writing – review and editing (equal). **catherine daniel:** Conceptualization (equal); data curation (equal); formal analysis (equal); funding acquisition (lead); investigation (lead); methodology (equal); project administration (lead); resources (equal); supervision (lead); writing – original draft (lead); writing – review and editing (lead).

## CONFLICT OF INTEREST

The authors declare that they have no conflicts of interest.

## Supporting information


Appendix S1

## Data Availability

All data generated or analysed during this study are included in this published article. Further inquiries can be directed to the corresponding author.
