# Supplementary material for: Study of the persistence and dynamics of recombinant mCherry‐producing Yarrowia lipolytica strains in the mouse intestine using fluorescence imaging
Source: Microb Biotechnol. 2022 Dec 20;16(3):618–31. doi: 10.1111/1751-7915.14178 (PMC9948224; doi:10.1111/1751-7915.14178)
Supplement: Supplementary file 1 — Appendix S1 [file MBT2-16-618-s001.docx]

Supplementary material


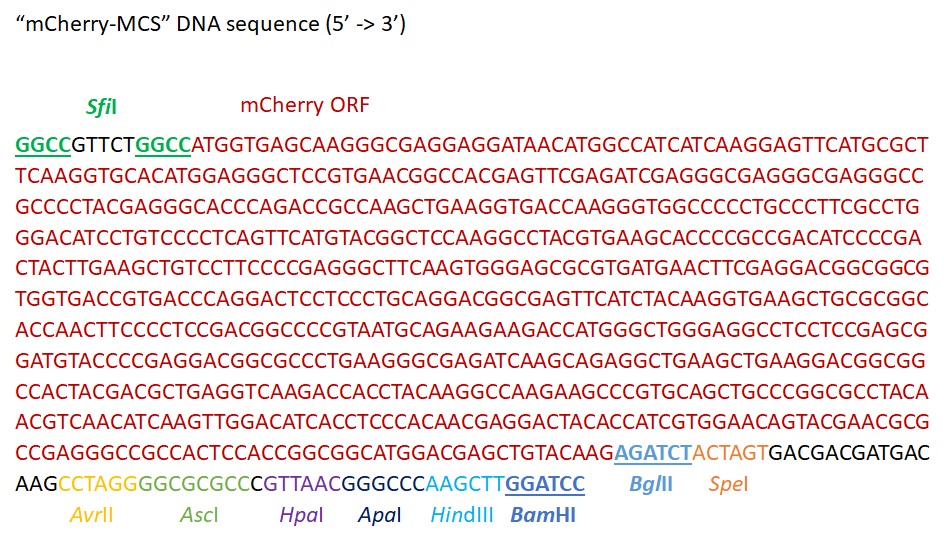


**Fig. S1 Sequence of the “mCherry-MCS” synthetic DNA fragment used to construct pINA1317-mCherry and pINA1317-mCherryMCS auto-cloning expression vectors.**

The “mCherry-MCS” fragment encompasses a *Sfi*I restriction site, the *mCherry* ORF (in red) and a MCS carrying 8 restriction sites: *Bgl*II, *Spe*I, *Avr*II, *Asc*I, *Hpa*I, *Apa*I, *Hin*dIII and *Bam*HI. A *Sfi*I-*Bgl*II sub-fragment, carrying the *mCherry* ORF only, was inserted between *Sfi*I and *Bam*HI (a site with *Bgl*II ligation-compatible overhangs) that are unique restriction sites of the pINA1317-YlCWP110 auto-cloning expression vector (Yue *et al*., 2008) to generate pINA1317-mCherry. A *Sfi*I-*Bam*HI sub-fragment, carrying the *mCherry* ORF fused to the MCS, was inserted between the *Sfi*I and *Bam*HI sites of pINA1317-YlCWP110 to generate pINA1317-mCherryMCS.


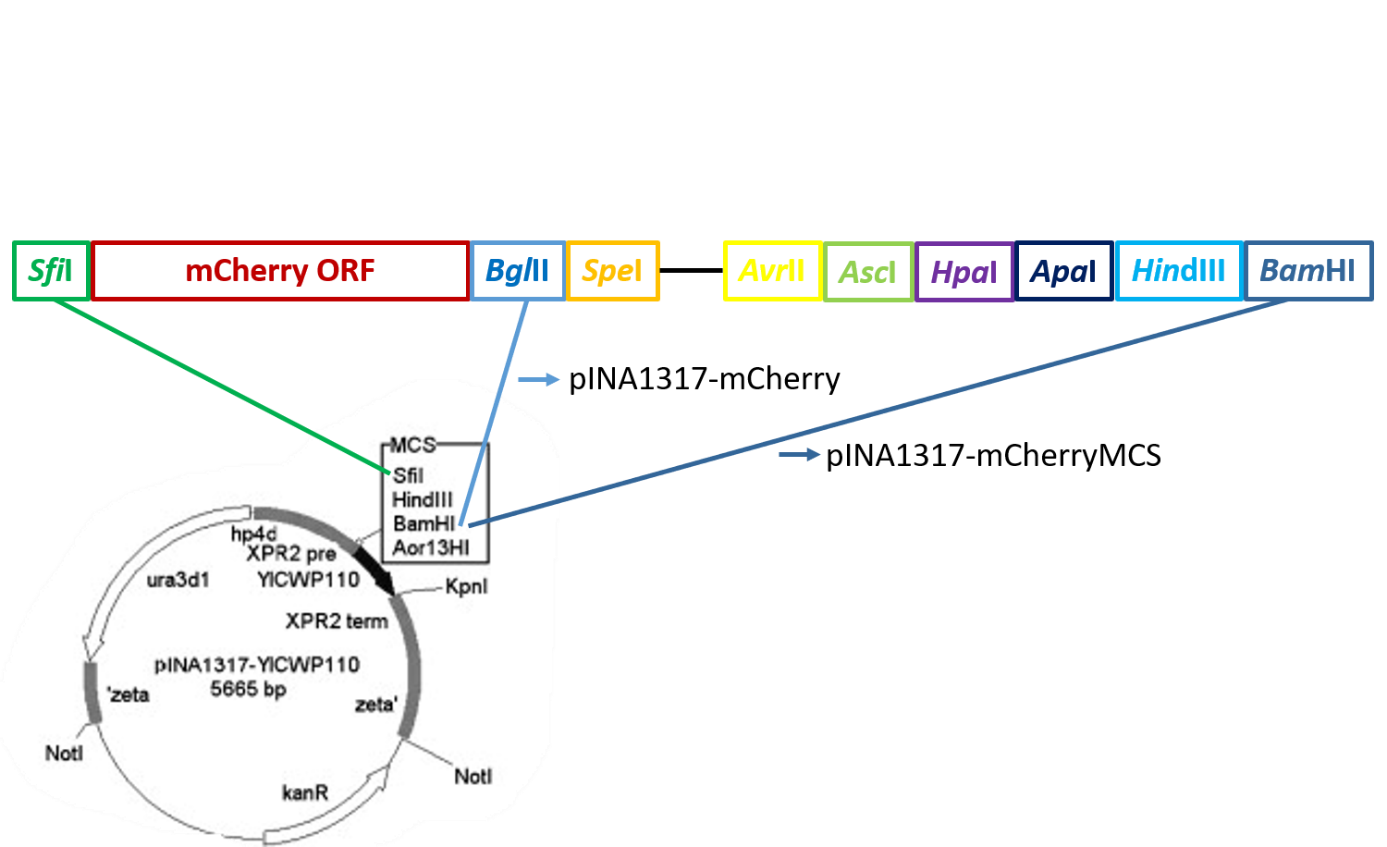


**Fig. S2 Cloning of “mCherry -MCS” in the pINA1317-YICWP110 expression vector**

The *Sfi*I-*Bgl*II and *Sfi*I-*Bam*HI DNA fragments from the “mCherry-MCS” fragment (schematized with the same color code as in Fig. S1) were cloned into the pINA1317-YlCWP110 expression vector, between the *Sfi*I and *Bam*HI sites (*Bgl*II and *Bam*HI having ligation-compatible overhangs) of the vector’s MCS. This resulted in the generation of plasmids pINA1317-mCherry and pINA1317-mCherryMCS, respectively. MCS, multiple cloning site.

**
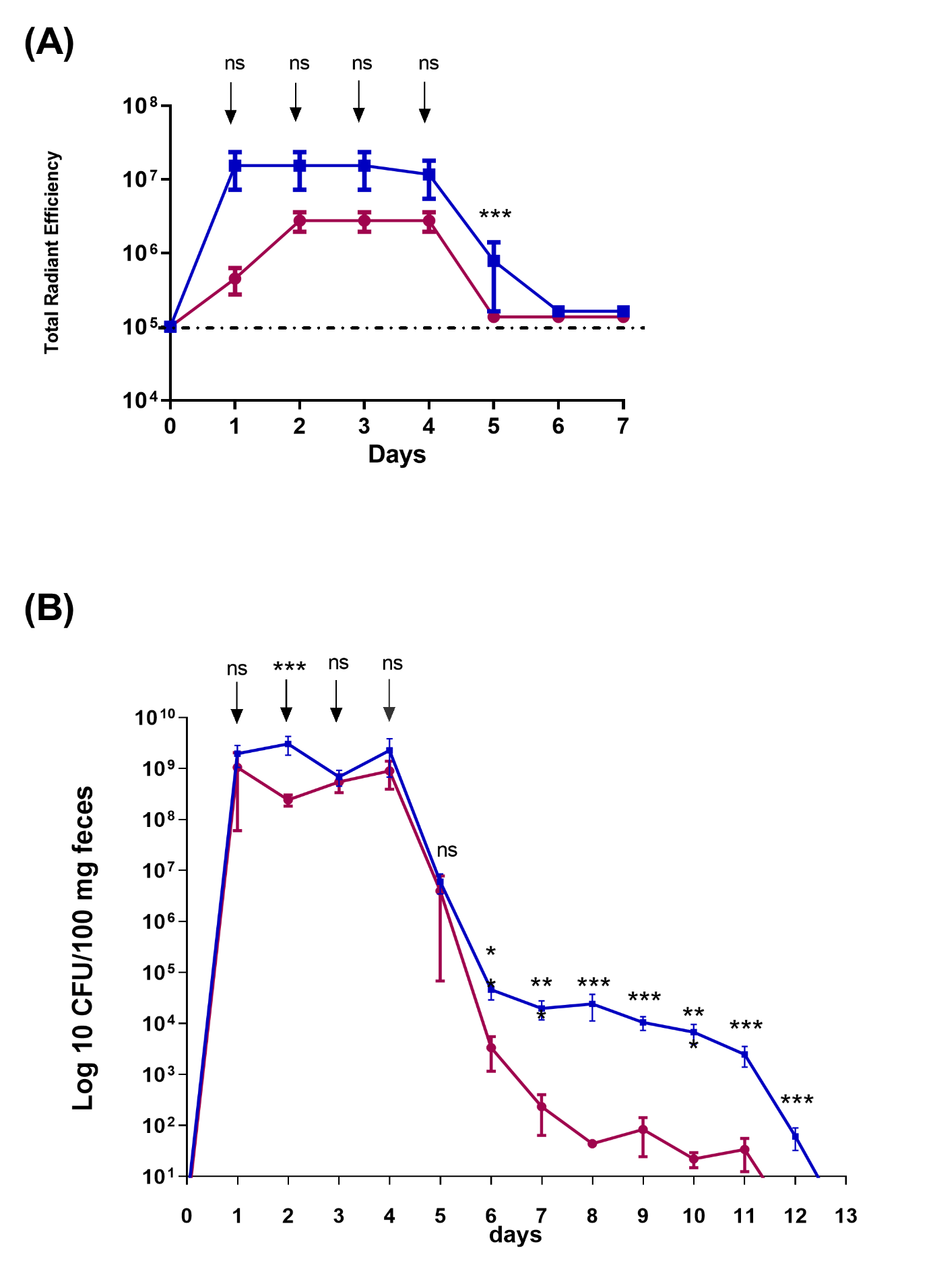
**

**Fig. S3. Persistence of m-Cherry producing *L. plantarum* (represented in blue) and *Y. lipolytica* 1E07 (represented in dark pink) in the feces of healthy mice after four daily oral administrations and quantification of their respective fluorescence signal.** Feces were collected daily from day 0 to day 13 (n=5 mice/group). A. Total radiant efficiency in [(p/s/cm2/sr)/(μW/cm2)] per 100 mg of feces with standard deviations. The background level for the fluorescent signal is represented by a dashed line. B. Averages of the daily CFU counts per 100 mg of feces with standard deviations. Overall differences between the two groups were assessed using the Kruskal-Wallis non-parametric test and those found to be significant are indicated with * (p<0.05), ** (p<0.01), *** and (p<0.001). ns: non-significant.


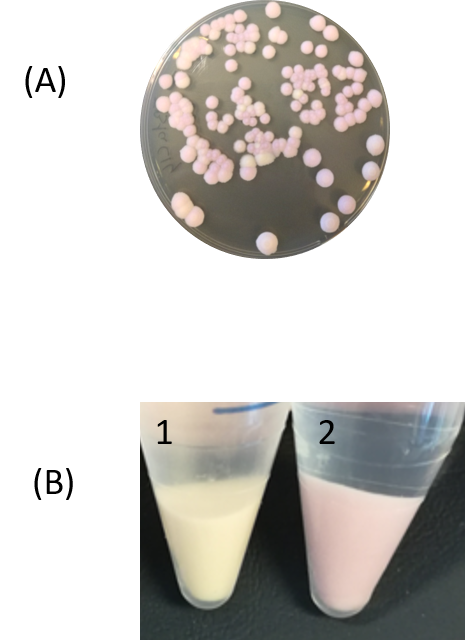


**Fig. S4. Yl1E07-mCherry pink representative colonies and cell pellets.** A. Transformation of 1E07-ura *Y. lipolytica* strain with pINA1317-mCherry plasmid exemplifying the pink coloration of transformant yeast colonies grown on YPD medium agar plates. B. Pink coloration of the cell pellet (obtained by centrifugation of an overnight culture in YPD liquid medium) from a selected Yl1E07-mCherry transformant (2) compared to the creamy coloration of that from the parent 1E07 strain (1)
